# Supplementary material for: Association of Low Handgrip Strength with Chemotherapy Toxicity in Digestive Cancer Patients: A Comprehensive Observational Cohort Study (FIGHTDIGOTOX)
Source: Nutrients. 2022 Oct 22;14(21):4448. doi: 10.3390/nu14214448 (PMC9654937; doi:10.3390/nu14214448)
Supplement: Supplementary file 1 [file nutrients-14-04448-s001.zip › nutrients-1955653-supplementary.pdf]

Supplementary Table S1. Population characteristics according to low and normal hand grip strength based on the median (exploratory low HGS).

Characteristics of patients	Level	Overall	Dynapenia[1]	Normal HGS[1]	*p*-value
Total, *n* (%)		244	107 (43.9)	137 (56.1)	
Sex, *n* (%)	Female	109 (44.7)	50 (46.7)	59 (43.1)	0.659
	Male	135 (55.3)	57 (53.3)	78 (56.9)	
Age, median (IQR)		69.0 (59.0-74.0)	72.0 (68.0 - 78.0)	63.0 (56.0 - 71.0)	<0.001
BMI, median (IQR)		24.6 (21.5-28.6)	24.9 (21.5 - 29.4)	24.3 (21.6 - 27.5)	0.522
ECOG PS, *n* (%)	0	66 (27.0)	20 (18.7)	46 (33.6)	0.006
	1	150 (61.5)	68 (63.6)	82 (59.9)	
	2	26 (10.7)	17 (15.9)	9 (6.6)	
	3	2 (0.8)	2 (1.9)		
Serum albumin level, median (IQR)		39.0 (36.0-42.0)	39.0 (34.0 - 41.5)	39.0 (37.0 - 42.0)	0.153
CRP, median (IQR)		9.0 (4.0-33.2)	11.0 (4.0 - 36.5)	8.0 (3.0-33.0)	0.121
mGPS, *n* (%)	0	118 (48.4)	46 (43.0)	72 (52.6)	0.020
	1	87 (35.7)	36 (33.6)	51 (37.2)	
	2	39 (16.0)	25 (23.4)	14 (10.2)	
Lymphopenia, *n* (%)	No	226 (92.6)	99 (92.5)	127 (92.7)	1.000
	Yes	18 (7.4)	8 (7.5)	10 (7.3)	
G8 score, median[2] (IQR)		12.0 (11.0-15.0)	12.0 (10.0 - 13.8)	13.0 (11.0-15.0)	0.050
Primary tumor location, *n* (%)	Colon and rectum	105 (43.2)	55 (51.4)	50 (36.8)	55 (51.4)
	Stomach	26 (10.7)	6 (5.6)	20 (14.7)	
	Esophagus	18 (7.4)	10 (9.3)	8 (5.9)	
	Pancreas	69 (28.4)	28 (26.2)	41 (30.1)	
	Others[3]	25 (10.2)	8 (7.5)	17 (12.5)	
Stage, *n* (%)	Localized	84 (34.4)	39 (36.4)	45 (32.8)	0.707
	Locally-advanced	57 (23.4)	26 (24.3)	31 (22.6)	
	Metastatic	103 (42.2)	42 (39.3)	61 (44.5)	
Number of metastatic sites, *n* (%)	1	68 (65.4)	28 (65.1)	40 (65.6)	1.000
	≥ 2	36 (34.6)	15 (34.9)	21 (34.4)	
Chemotherapy regimen, *n* (%)	5FU + Oxaliplatin	96 (39.3)	46 (43.0)	50 (36.5)	0.072
	5FU + Irinotecan + Oxaliplatin	69 (28.3)	22 (20.6)	47 (34.3)	
	5FU alone	24 (9.8)	15 (14.0)	9 (6.6)	
	Gemcitabine	18 (7.4)	9 (8.4)	9 (6.6)	
	Others[4]	37 (15.1)	15 (14)	22 (16.1)	
Biotherapy, *n* (%)	None	204 (83.6)	89 (83.2)	115 (83.9)	0.036
	Bevacizumab	26 (10.7)	16 (15.0)	10 (7.3)	
	Others [5]	14 (5.7)	2 (1.8)	12 (8.8)	

Concomitant radiotherapy, *n* (%)	No	222 (91.0)	99 (92.5)	123 (89.8)	0.605
	Yes	22 (9.0)	8 (7.5)	14 (10.2)	

Abbreviations: 5FU: 5 Fluorouracil; BMI: Body Mass Index; CRP: C-reactive protein; ECOG PS: Eastern Cooperative Oncology Group Criteria Performance Status; HGS: handgrip strength; IQR: interquartile range; mGPS: modified Glasgow prognosis score.

[1] According HGS cut-off based on the median in the population as HGS <34 for men and <22kg for women; [2] Data available for 82 patients; [3] Other localizations: biliary tract (*n*=8), small intestine (*n*=7), ampulla of Vater (*n*=3), neuroendocrine tumor (*n*=4), appendix (*n*=1), anal (*n*=1), unknown primary (*n*=1); [4] Other chemotherapy: 5FU + Irinotecan (*n*=8), 5FU + Oxaliplatin + Docetaxel (*n*=9), 5FU + Cisplatin (*n*=1), 5FU + Dacarbazine (*n*=3), Carboplatin-Etoposide (*n*=1), Gemcitabine + Cisplatin (*n*=4), Gemcitabine + Oxaliplatin (*n*=3), Capecitabine + Oxaliplatin (*n*=7), Capecitabine + Mitomycin (*n*=1); [5] Other biotherapy: Panitumumab (*n*=10), Trastuzumab (*n* =4).

Supplementary Table S2. Association between low hand grip strength based on the median (exploratory low HGS) and chemotherapy-induced toxicities and DLT (detailed grades and therapeutic modifications).

	Overall (n = 244)	Dynapenia[1] (n = 23)	Normal HGS[1] (n = 221)	p value
**Neuropathy**				
Neuropathy (all grade), n (%)	174 (94.6)	66 (91.7)	108 (96.4)	0.193
DLT, n (%)	76 (41.3)	26 (36.1)	50 (44.6)	0.285
Toxicity grade 1, n (%)	103 (58.9)	43 (64.2)	60 (55.6)	0.334
Toxicity grade 2, n (%)	61 (34.9)	19 (28.4)	42 (38.9)	
Toxicity grade 3, n (%)	11 (6.3)	5 (7.5)	6 (5.6)	
Median appearance, days (IQR)	28.0 (14.0 - 42.0)	28.0 (14.0 - 42.0)	28.0 (14.0 - 42.2)	0.619
Decrease of dose, n (%)	26 (14.1)	12 (16.7)	14 (12.5)	0.717
Discontinuation, n (%)	64 (34.8)	19 (26.4)	45 (40.2)	0.152
Delay of chemotherapy, n (%)	0			
**Asthenia**				
Toxicity (all grade), n (%)	224 (91.8)	104 (97.2)	120 (87.6)	0.014
DLT, n (%)	24 (9.8)	16 (15.0)	8 (5.8)	0.029
Toxicity grade 1, n (%)	145 (64.4)	59 (56.7)	86 (71.1)	0.071
Toxicity grade 2, n (%)	57 (25.3)	31 (29.8)	26 (21.5)	
Toxicity grade 3, n (%)	23 (10.2)	14 (13.5)	9 (7.4)	
Median appearance, days (IQR)	17.0 (14.0 - 39.0)	17.0 (14.0 - 29.2)	17.0 (14.0 - 42.0)	0.687
Decrease of dose, n (%)	15 (6.1)	9 (8.4)	6 (4.4)	0.002
Discontinuation, n (%)	7 (2.9)	5 (4.7)	2 (1.5)	0.001
Delay of chemotherapy, n (%)	13 (5.3)	9 (8.4)	4 (2.9)	0.001
Diarrhea				
Toxicity (all grade), n (%)	139 (57.0)	56 (52.3)	83 (60.6)	0.214
DLT, n (%)	20 (8.2)	9 (8.4)	11 (8.0)	1.000
Toxicity grade 1, n (%)	82 (59.4)	25 (44.6)	57 (69.5)	0.015
Toxicity grade 2, n (%)	40 (29.0)	22 (39.3)	18 (22.0)	
Toxicity grade 3, n (%)	15 (10.9)	8 (14.3)	7 (8.5)	
Toxicity grade 4, n (%)	1 (0.7)	1 (1.8)	0	
Median appearance, days (IQR)	16.0 (14.0 - 42.0)	27.0 (14.0 - 42.0)	15.0 (14.0 - 40.0)	0.293
Decrease of dose, n (%)	13 (5.3)	5 (4.7)	8 (5.8)	0.550
Discontinuation, n (%)	6 (2.5)	4 (3.7)	2 (1.5)	0.242
Delay of chemotherapy, n (%)	7 (2.9)	4 (3.7)	3 (2.2)	0.324
**Nausea[3]**				
Toxicity (all grade), n (%)	115 (56.9)	41 (49.4)	74 (62.2)	0.084

DLT, *n* (%)	4 (2.0)	2 (2.4)	2 (1.7)	1.000
Toxicity grade 1, *n* (%)	76 (66.1)	24 (58.5)	52 (70.3)	0.437
Toxicity grade 2, *n* (%)	35 (30.4)	15 (36.6)	20 (27.0)	
Toxicity grade 3, *n* (%)	4 (3.5)	2 (4.9)	2 (2.7)	
Median appearance, days (IQR)	15.0 (14.0 - 33.0)	28.0 (14.0 - 42.0)	14.5 (14.0 - 28.0)	0.115
Decrease of dose, *n* (%)	4 (2.0)	2 (2.4)	2 (1.7)	0.135
Discontinuation, *n* (%)	1 (0.5)	1 (1.2)	0	0.043
Delay of chemotherapy, *n* (%)	1 (0.5)	0	1 (0.8)	0.110
**Vomiting**[3]				
Toxicity (all grade), *n* (%)	51 (25.2)	15 (18.1)	36 (30.3)	0.047
DLT, *n* (%)	4 (2.0)	1 (1.2)	3 (2.5)	0.645
Toxicity grade 1, *n* (%)	35 (67.3)	9 (60.0)	26 (70.3)	0.775
Toxicity grade 2, *n* (%)	14 (26.9)	5 (33.3)	9 (24.3)	
Toxicity grade 3, *n* (%)	3 (5.8)	1 (6.7)	2 (5.4)	
Median appearance, days (IQR)	38.0 (15.0 - 77.2)	42.0 (15.0 - 74.0)	28.0 (15.0 - 77.0)	0.871
Decrease of dose, *n* (%)	2 (1.0)	0	2 (1.7)	0.034
Discontinuation, *n* (%)	2 (1.0)	0	2 (1.7)	0.034
Delay of chemotherapy, *n* (%)	2 (1.0)	1 (1.2)	1 (0.8)	0.030
**Neutropenia**				
Toxicity (all grade), *n* (%)	60 (24.6)	26 (24.3)	34 (24.8)	1.000
DLT, *n* (%)	28 (11.5)	10 (9.3)	18 (13.1)	0.172
Toxicity grade 1, *n* (%)	11 (18.0)	7 (26.9)	4 (11.4)	0.086
Toxicity grade 2, *n* (%)	21 (34.4)	6 (23.1)	15 (42.9)	
Toxicity grade 3, *n* (%)	24 (39.3)	9 (34.6)	15 (42.9)	
Toxicity grade 4, *n* (%)	5 (8.2)	4 (15.4)	1 (2.9)	
Median appearance, days (IQR)	37.5 (23.8 - 68.0)	30.0 (19.2 - 51.0)	41.5 (27.0 - 90.5)	0.108
Discontinuation, *n* (%)	1 (0.4)	0	1 (0.7)	1.000
Delay of chemotherapy, *n* (%)	20 (8.2)	8 (7.5)	12 (8.8)	0.901
**Anemia**				
Toxicity (all grade), *n* (%)	165 (67.6)	83 (77.6)	82 (59.9)	0.006
DLT, *n* (%)	6 (2.5)	4 (3.7)	2 (1.5)	0.409
Toxicity grade 1, *n* (%)	102 (61.8)	48 (57.8)	54 (65.9)	0.345
Toxicity grade 2, *n* (%)	48 (29.1)	25 (30.1)	23 (28.0)	
Toxicity grade 3, *n* (%)	15 (9.1)	10 (12.0)	5 (6.1)	
Median appearance, days (IQR)	15.0 (13.0 - 41.0)	14.0 (13.0 - 29.5)	26.5 (14.0 - 54.5)	0.005
Decrease of dose, *n* (%)	6 (2.5)	4 (3.7)	2 (1.5)	0.012

Discontinuation, *n* (%)	0			
Delay of chemotherapy, *n* (%)	0			
**Thrombopenia**				
Toxicity (all grade), *n* (%)	73 (29.9)	32 (29.9)	41 (29.9)	1.000
DLT, *n* (%)	13 (5.3)	5 (4.7)	8 (5.8)	0.779
Toxicity grade 1, *n* (%)	58 (79.5)	27 (84.4)	31 (75.6)	0.288
Toxicity grade 2, *n* (%)	12 (16.4)	3 (9.4)	9 (22.0)	
Toxicity grade 3, *n* (%)	3 (4.1)	2 (6.2)	1 (2.4)	
Median appearance, days (IQR)	53.0 (27.0 - 90.0)	43.0 (25.2 - 71.5)	60.0 (28.0 - 98.0)	0.161
Decrease of dose, *n* (%)	4 (1.6)	1 (0.9)	3 (2.2)	0.805
Discontinuation, *n* (%)	1 (0.4)	4 (3.7)	1 (0.7)	0.937
Delay of chemotherapy, *n* (%)	8 (3.3)	4 (3.7)	4 (2.9)	0.969
**Oral Mucositis**[4]				
Toxicity (all grade), *n* (%)	29 (11.9)	15 (14.0)	14 (10.2)	0.455
DLT, *n* (%)	3 (1.2)	1 (0.9)	2 (1.5)	1.000
Toxicity grade 1, *n* (%)	22 (78.6)	13 (86.7)	9 (69.2)	0.533
Toxicity grade 2, *n* (%)	3 (10.7)	1 (6.7)	2 (15.4)	
Toxicity grade 3, *n* (%)	3 (10.7)	1 (6.7)	2 (15.4)	
Decrease of dose, *n* (%)	3 (1.2)	1 (0.9)	2 (1.5)	0.525
Discontinuation, *n* (%)	0			
Delay of chemotherapy, *n* (%)	0			
**Hand Foot Syndrome**[4]				
Toxicity (all grade), *n* (%)	10 (4.8)	5 (5.4)	5 (4.3)	0.629
DLT, *n* (%)	4 (1.9)	2 (2.2)	2 (1.7)	1.000
Toxicity grade 1, *n* (%)	3 (30.0)	2 (40.0)	1 (20.0)	0.282
Toxicity grade 2, *n* (%)	5 (50.0)	3 (60.0)	2 (40.0)	
Toxicity grade 3, *n* (%)	2 (20.0)	0	2 (40.0)	
Median appearance, days (IQR)	53.0 (40.0 - 68.8)	54.0 (37.0 - 70.0)	52.0 (43.0 - 65.0)	0.917
Decrease of dose, *n* (%)	3 (1.4)	1 (1.1)	2 (1.7)	0.733
Discontinuation, *n* (%)	1 (0.5)	1 (1.1)	0	0.531
Delay of chemotherapy, *n* (%)	0			

Abbreviation: DLT: Dose Limiting Toxicity; HGS: Hand Grip Strength; IQR: Interquartile
[1] HGS cut-off based on the median in the population as HGS <34 for men and <22kg for women (exploratory low HGS) ; [2] Only patients receiving neurotoxic chemotherapy (*n*=184), and graded according Levi scale ; [3] Patients receiving 5FU and gemcitabine alone were not analyzed for this adverse effect (*n*=202) ; [4] Only patients receiving 5FU- or capecitabine-based chemotherapy regimen (*n*=210).

Supplementary Table S3. Association between dynapenia based on EWSGOP2 criteria and chemotherapy-induced toxicities and DLT (detailed grades and therapeutic modifications).

	Overall (*n* = 244)	Dynapenia[1] (*n* = 23)	Normal HGS[1] (*n* = 221)	*p* value
**Neuropathy[2]**				
Neuropathy (all grade), *n* (%)	174 (94.6)	14 (93.3)	160 (94.7)	0.582
DLT, *n* (%)	76 (41.3)	7 (46.7)	69 (40.8)	0.786
Toxicity grade 1, *n* (%)	103 (58.9)	8 (53.3)	95 (59.4)	0.116
Toxicity grade 2, *n* (%)	61 (34.9)	4 (26.7)	57 (35.6)	
Toxicity grade 3, *n* (%)	11 (6.3)	3 (20.0)	8 (5.0)	
Median appearance, days (IQR)	28.0 (14.0 - 42.0)	18.0 (14.0 - 42.0)	28.0 (14.0 - 42.0)	0.274
Decrease of dose, *n* (%)	26 (14.1)	6 (40.0)	20 (11.8)	0.025
Discontinuation, *n* (%)	64 (34.8)	3 (20.0)	61 (36.1)	0.335
Delay of chemotherapy, *n* (%)	0			
**Asthenia**				
Toxicity (all grade), *n* (%)	224 (91.8)	23 (100.0)	201 (91.0)	0.303
DLT, *n* (%)	24 (9.8)	5 (21.7)	19 (8.6)	0.059
Toxicity grade 1, *n* (%)	145 (64.4)	11 (47.8)	134 (66.3)	0.156
Toxicity grade 2, *n* (%)	57 (25.3)	8 (34.8)	49 (24.3)	
Toxicity grade 3, *n* (%)	23 (10.2)	4 (17.4)	19 (9.4)	
Median appearance, days (IQR)	17.0 (14.0 - 39.0)	15.0 (14.0 - 28.0)	19.0 (14.0 - 40.8)	0.469
Decrease of dose, *n* (%)	15 (6.1)	3 (13.0)	12 (5.4)	0.093
Discontinuation, *n* (%)	7 (2.9)	2 (8.7)	5 (2.3)	0.062
Delay of chemotherapy, *n* (%)	13 (5.3)	3 (13.0)	10 (4.5)	0.071
**Diarrhea**				
Toxicity (all grade), *n* (%)	139 (57.0)	12 (52.2)	127 (57.5)	0.693
DLT, *n* (%)	20 (8.2)	2 (8.7)	18 (8.1)	1.000
Toxicity grade 1, *n* (%)	82 (59.4)	5 (41.7)	77 (61.1)	0.071
Toxicity grade 2, *n* (%)	40 (29.0)	4 (33.3)	36 (28.6)	
Toxicity grade 3, *n* (%)	15 (10.9)	2 (16.7)	13 (10.3)	
Toxicity grade 4, *n* (%)	1 (0.7)	1 (8.3)		
Median appearance, days (IQR)	16.0 (14.0 - 42.0)	20.0 (14.0 - 29.0)	15.0 (14.0 - 42.0)	0.915
Decrease of dose, *n* (%)	13 (5.3)	0	13 (5.9)	0.760
Discontinuation, *n* (%)	6 (2.5)	1 (4.3)	5 (2.3)	0.482
Delay of chemotherapy, *n* (%)	7 (2.9)	1 (4.3)	6 (2.7)	0.367
**Nausea[3]**				
Toxicity (all grade), *n* (%)	115 (56.9)	9 (52.9)	106 (57.3)	0.801

DLT, *n* (%)	4 (2.0)	1 (5.9)	3 (1.6)	0.298
Toxicity grade 1, *n* (%)	76 (66.1)	6 (66.7)	70 (66.0)	0.380
Toxicity grade 2, *n* (%)	35 (30.4)	2 (22.2)	33 (31.1)	
Toxicity grade 3, *n* (%)	4 (3.5)	1 (11.1)	3 (2.8)	
Median appearance, days (IQR)	15.0 (14.0 - 33.0)	16.0 (15.0 - 42.0)	15.0 (14.0 - 29.0)	0.561
Decrease of dose, *n* (%)	4 (2.0)	1 (5.9)	3 (1.6)	0.283
Discontinuation, *n* (%)	1 (0.5)	0	1 (0.5)	0.818
Delay of chemotherapy, *n* (%)	1 (0.5)	0	1 (0.5)	0.818
**Vomiting**[3]				
Toxicity (all grade), *n* (%)	51 (25.2)	3 (17.6)	48 (25.9)	0.605
DLT, *n* (%)	4 (2.0)	0	4 (2.2)	1.000
Toxicity grade 1, *n* (%)	35 (67.3)	1 (33.3)	34 (69.4)	0.102
Toxicity grade 2, *n* (%)	14 (26.9)	1 (33.3)	13 (26.5)	
Toxicity grade 3, *n* (%)	3 (5.8)	1 (33.3)	2 (4.1)	
Median appearance, days (IQR)	38.0 (15.0 - 77.2)	39.0 (27.0 - 48.5)	37.0 (15.0 - 78.0)	0.921
Decrease of dose, *n* (%)	2 (1.0)	0	2 (1.1)	0.637
Discontinuation, *n* (%)	2 (1.0)	0	2 (1.1)	0.637
Delay of chemotherapy, *n* (%)	2 (1.0)	0	2 (1.1)	0.637
**Neutropenia**				
Toxicity (all grade), *n* (%)	60 (24.6)	3 (13.0)	57 (25.8)	0.286
DLT, *n* (%)	28 (11.5)	0	28 (12.7)	0.086
Toxicity grade 1, *n* (%)	11 (18.0)	2 (66.7)	9 (15.5)	0.028
Toxicity grade 2, *n* (%)	21 (34.4)	0	21 (36.2)	
Toxicity grade 3, *n* (%)	24 (39.3)	0	24 (41.4)	
Toxicity grade 4, *n* (%)	5 (8.2)	1 (33.3)	4 (6.9)	
Median appearance, days (IQR)	37.5 (23.8 - 68.0)	26.0 (23.0 - 28.5)	39.0 (25.0 - 68.0)	0.277
	11 (4.5)	0	11 (5.0)	0.522
Discontinuation, *n* (%)	20 (8.2)	0	20 (9.0)	0.373
Delay of chemotherapy, *n* (%)	1 (0.4)	0	1 (0.5)	0.369
**Anemia**				
Toxicity (all grade), *n* (%)	165 (67.6)	19 (82.6)	146 (66.1)	0.238
DLT, *n* (%)	6 (2.5)	1 (4.3)	5 (2.3)	0.451
Toxicity grade 1, *n* (%)	102 (61.8)	13 (68.4)	89 (61.0)	0.695
Toxicity grade 2, *n* (%)	48 (29.1)	4 (21.1)	44 (30.1)	
Toxicity grade 3, *n* (%)	15 (9.1)	2 (10.5)	13 (8.9)	
Median appearance, days (IQR)	15.0 (13.0 - 41.0)	14.0 (14.0 - 41.0)	15.5 (13.0 - 41.0)	0.912

Decrease of dose, *n* (%)	6 (2.5)	1 (4.3)	5 (2.3)	0.189
Discontinuation, *n* (%)	0			
Delay of chemotherapy, *n* (%)	0			
**Thrombopenia**				
Toxicity (all grade), *n* (%)	73 (29.9)	10 (43.5)	63 (28.5)	0.235
DLT, *n* (%)	13 (5.3)	2 (8.7)	11 (5.0)	0.352
Toxicity grade 1, *n* (%)	58 (79.5)	8 (80.0)	50 (79.4)	0.591
Toxicity grade 2, *n* (%)	12 (16.4)	1 (10.0)	11 (17.5)	
Toxicity grade 3, *n* (%)	3 (4.1)	1 (10.0)	2 (3.2)	
Median appearance, days (IQR)	53.0 (27.0 - 90.0)	55.5 (31.2 - 85.8)	52.0 (27.0 - 88.0)	0.810
Decrease of dose, *n* (%)	4 (1.6)	0	4 (1.8)	0.201
Discontinuation, *n* (%)	1 (0.4)	0	1 (0.5)	0.227
Delay of chemotherapy, *n* (%)	8 (3.3)	2 (8.7)	6 (2.7)	0.121
**Oral Mucositis**[4]				
Toxicity (all grade), *n* (%)	29 (11.9)	2 (8.7)	27 (12.2)	0.836
DLT, *n* (%)	3 (1.2)	0	3 (1.4)	1.000
Toxicity grade 1, *n* (%)	22 (78.6)	2 (100.0)	20 (76.9)	0.745
Toxicity grade 2, *n* (%)	3 (10.7)	0	3 (11.5)	
Toxicity grade 3, *n* (%)	3 (10.7)	0	3 (11.5)	
Decrease of dose, *n* (%)	3 (1.2)	0	3 (1.4)	0.805
Discontinuation, *n* (%)	0			
Delay of chemotherapy, *n* (%)	0			
**Hand Foot Syndrome**[4]				
Toxicity (all grade), *n* (%)	10 (4.8)	4 (18.2)	6 (3.2)	0.007
DLT, *n* (%)	4 (1.9)	2 (9.1)	2 (1.1)	0.075
Toxicity grade 1, *n* (%)	3 (30.0)	1 (25.0)	2 (33.3)	0.329
Toxicity grade 2, *n* (%)	5 (50.0)	3 (75.0)	2 (33.3)	
Toxicity grade 3, *n* (%)	2 (20.0)	0	2 (33.3)	
Median appearance, days (IQR)	53.0 (40.0 - 68.8)	62.0 (49.8 - 96.2)	47.5 (40.0 - 61.8)	0.394
Decrease of dose, *n* (%)	3 (1.4)	1 (4.5)	2 (1.1)	0.007
Discontinuation, *n* (%)	1 (0.5)	1 (4.5)	0	0.001
Delay of chemotherapy, *n* (%)	0			

Abbreviation: DLT: Dose Limiting Toxicity; HGS: Hand Grip Strength; IQR: Interquartile
[1] Dynapenia was defined according the EWGSOP2 as HGS <27kg for men and <16kg for women; [2] Only patients receiving neurotoxic chemotherapy (*n*=184), and graded according to Levi scale; [3] Patients receiving 5FU and gemcitabine alone were not analyzed for this adverse effect (*n*=202); [4] Only patients receiving 5FU- or capecitabine-based chemotherapy regimen (*n*=210).
